# Towards a practical threat assessment methodology for crop landraces

**DOI:** 10.3389/fpls.2024.1336876

**Published:** 2024-02-22

**Authors:** Maria João Almeida, Ana Maria Barata, Stef De Haan, Bal Krishna Joshi, Joana Magos Brehm, Mariana Yazbek, Nigel Maxted

**Affiliations:** ^1^ School of Biosciences, University of Birmingham, Birmingham, United Kingdom; ^2^ Banco Português de Germoplasma Vegetal, Instituto Nacional de Investigação Agrária e Veterinária, Braga, Portugal; ^3^ Andean Food Systems, International Potato Center, Lima, Peru; ^4^ National Gene Bank, National Agricultural Research Centre, Kathmandu, Nepal; ^5^ Genebank, International Center for Agricultural Research in the Dry Areas, Terbol, Lebanon

**Keywords:** conservation, crop landraces, extinction, genetic erosion, methodology, plant genetic resources, threat assessment

## Abstract

Crop landraces (LR), the traditional varieties of crops that have been maintained for millennia by repeated cycles of planting, harvesting, and selection, are genetically diverse compared to more modern varieties and provide one of the key components for crop improvement due to the ease of trait transfer within the crop species. However, LR diversity is increasingly threatened with genetic erosion and extinction by replacement with improved cultivars, lack of incentives for farmers to maintain traditional agricultural systems, and rising threats from climate change. Their active conservation is necessary to maintain this critical resource. However, as there are hundreds of thousands of LR and millions of LR populations for crops globally, active conservation is complex and resource-intensive. To assist in implementation, it is useful to be able to prioritise LR for conservation action and an obvious means of prioritisation is based on relative threat assessment. There have been several attempts to propose LR threat assessment methods, but none thus far has been widely accepted or applied. The aim of this paper is to present a novel, practical, standardised, and objective methodology for LR threat assessment derived from the widely applied IUCN Red Listing for wild species, involving the collation of time series information for LR population range, LR population trend, market, and farmer characteristics and LR context information. The collated information is compared to a set of threat criteria and an appropriate threat category is assigned to the LR when a threshold level is reached. The proposed methodology can be applied at national, regional, or global levels and any crop group.

## 1 Introduction

Globally, 135 million people in 2019 from 55 countries were reported to be facing phase 3 Crisis level food insecurity or worse, which is a 60% increase compared to 2015 when the figure was 80 million, while in total about 850 million people in the world were undernourished in 2021 (FAO et al., 2021). The human population is today 8.09 billion (22^nd^ May 2023) and is predicted to rise to 9.7 billion by 2050, with 86% in developing countries (United Nations, 2021). It is predicted that global food production will need to grow by 60% globally, and 100% in developing countries compared to the 2005/2007 production levels to meet this growing demand (FAO, 2011). At the same time, crop production may decrease by 2% per decade if crop varieties are not adapted to the changing environment (IPCC, 2014). Although there are political reasons for food shortages, there are issues of food wastage and post-harvest losses to consider, plant breeders are increasingly requiring novel genetic diversity to increase production (Litrico and Violle, 2015). This diversity is often found in the traditionally grown, genetically diverse crop landraces (LR), which have not been bred for trait uniformity like modern cultivars.

Camacho-Villa et al. (2005) defined an LR as “a dynamic population(s) of a cultivated plant that has historical origin, distinct identity and lacks formal crop improvement, as well as often being genetically diverse, locally adapted and associated with traditional farming systems”. The importance of the utilisation of LR is well recognised, as they often contain unique trait diversity due to their adaption to the location where they developed, and trait introgression is relatively easy compared to crop wild relatives as there is no crossing barrier and they do not, through linkage drag, bring deleterious alleles that need to be excluded (Ellstrand, 2003). This adaptive trait diversity can sustain yield for LR in marginal environments and mitigate diseases or pest attacks, as well as drought, frost, and salinity tolerance, and even yield enhancement in improved varieties (Harlan, 1975; Frankel et al., 1995; Veteläinen et al., 2009). Importantly, LRs are often maintained by smallholders and indigenous farmers because of the multiple cultural, provisioning, and regulating ecosystem services they provide (de Haan, 2021). These may include diverse benefits such as cultural and local identities, superior organoleptic properties, and relative yield stability in marginal and/or variable environments, among other factors (Perales et al., 2005; Fliedel et al., 2013; Ortman et al., 2023).

Regardless of their obvious economic value, it is well established that LR are increasingly, globally threatened (Vavilov, 1957; Bennett, 1971; 1973; Harlan, 1972; 1975; Frankel, 1970; Frankel, 1972; Frankel, 1973; Hawkes, 1983) and it has been argued that they are the most severely threatened element of all biodiversity (Maxted, 2006). The justification for this proposition being: (i) there are very few inventories of extant LR in each country, each region, or globally (Maxted and Scholten, 2007; FAO, 2011: Jarvis et al., 2011; de Boef et al., 2013; Almeida et al., 2023); (ii) some government agencies and seed companies are actively promoting the replacement of genetically diverse LR by modern genetically uniform cultivars (Frankel and Hawkes, 1975; Harlan, 1975; Negri, 2005); (iii) in most countries no agency is direct responsibility for their conservation (Raggi et al., 2022); (iv) LR sales have been and are impacted by seed legislation that requires all crop seed to be registered before it can be sold and to comply involves an additional cost to individual growers so inadvertently restricts seed sale and LR production (Maxted et al., 2013); (v) the internationalisation of food systems and pressure of evolving markets predicates varietal standards and uniformity (Negri, 2003; Joshi et al., 2004; Maxted et al., 2013); (vi) LR maintainers are often subsistence farmer growing LR for family or local consumption, but their prime motivation is commercial gain or food production not LR conservation for its own sake (Veteläinen et al., 2009); (vii) LR maintainers are almost always elderly and their number is dwindling each year (average age in the UK was 65 (Scholten et al., 2008); (viii) there is ineffective transmission of LR knowledge (cultivation and marketing) from maintainer generation to generation (Negri, 2003; Camacho-Villa et al., 2005); (ix) the traditional LR maintenance from generation to generation is breaking down with the children of maintainers failing to take over LR maintenance or farming altogether (Negri, 2003); (x) LR maintainers, like other rural populations globally are increasingly migrating from rural areas to cities and LR are often lost (Negri, 2005); and finally, (xi) there is the predicted detrimental impact of climate change on LR diversity (Jarvis et al., 2010). Each of these factors is threatening current LR diversity, both in terms of genetic (Hammer et al., 1996) and cultural/heritage (Negri, 2005) diversity loss and so inevitably likely to negatively impact future food security.

Effective conservation requires planning, which often includes conservation target prioritisation as conservation resources are always too limited to conserve all potential targets simultaneously (Kell et al., 2017). One commonly applied means of prioritisation is relative threat assessment, assessing the relative risk of extinction among competing conservation targets (Maxted et al., 2013). For wild species, the International Union for Conservation of Nature (IUCN) Categories and Criteria are universally recognised and used for threat assessment (IUCN, 2012). However, applying or adapting the IUCN Categories and Criteria for use in LR threat assessment is problematic because (a) it is not species or taxon threat assessment but genetic diversity within species or taxa that are being assessed for LR, (b) LR are crops that have been domesticated and therefore have intrinsically less genetic diversity than wild species, (c) LR populations are always managed by humans and local human management practices and global policies will impact LR maintenance and these factors must also be considered in LR assessment, and (d) government and industrial policies may encourage the promotion of high yielding cultigens and hybrid varieties that replace LR cultivation so actively eradicating LR diversity, such systematic eradication of wild species does not occur. Therefore, it is not feasible to use the standard IUCN Red List approach to LR threat assessment.

However, there is still the requirement for an effective means of LR threat assessment to focus conservation targeting and proposals have been made for LR threat assessment techniques (e.g., Joshi et al., 2004; Porfiri et al., 2009; Padulosi and Dulloo, 2012; de Haan et al., 2016), although no standardised LR threat assessment methodology is currently widely accepted or easily applied. Joshi et al. (2004) proposed categorising LR based on population, ecological, and social criteria (adapted from Brush, 2000), along with use and modernisation criteria, and there are obvious parallels to the IUCN categories. Hammer and Khoshbakht (2005) developed a list of threatened crop species not LR by correlating the IUCN Red List of Threatened Plants (Walter and Gillett, 1998) results with the list in the 3^rd^ edition of Mansfeld’s Encyclopaedia of Agricultural and Horticultural Crops (Hanelt and IPK, 2001). To rationally apply regional funds for sustaining landrace cultivation, Porfiri et al. (2009) assessed LR threat level using five criteria: (i) presence of the product on the market, (ii) presence in the catalogues of seed companies/nurseries, (iii) number of cultivating farmers, (iv) areas under cultivation (as a percentage of the total regional area for the species), (v) new dedicated area trend (presence of new areas reserved to LR cultivation). Antofie et al. (2010) extended the work of Hammer (1991), and Hammer and Khoshbakht (2005) and suggested adapting the Red Listing approach for LR. The authors produced a data sheet for each LR including crop and LR vernacular and scientific names; seed origin; cultivation and location details; conservation status; photographs; authors and references. The data sheet presented information that would help identify LR Red Lists but did not actually assess individual LR threat. Voegel (2012) advocated using diverse crop information (e.g., historical material; statistical registers; lists/inventories of cultivars; scientific literature) to formulate a Red List system, based on the continuity of cultivation and use of a crop and cultivars over time in a certain location. Further in the same year, Padulosi and Dulloo (2012) proposed creating a Red List of cultivated plant species/varieties based on five steps: i) General assessment and inventory of LR; ii) Red List and vulnerable variety list establishment; iii) First validation of Red Lists; iv) Second validation of Red Lists; and v) Documentation and monitoring. More recently de Haan et al. (2016); de Haan et al. (2019), stress the importance of time series data in LR population monitoring, they suggest using (i) hotspot identification, (ii) total diversity, (iii) relative diversity, (iv) spatial diversity, and (v) collective knowledge as indicators of threat. Despite the individual merits of each of these approaches and their evolving refinement over time, most do not fully address the requirement to assess LR infra-specific level of threat, nor have they been widely applied by the global agrobiodiversity community. Also, the lack of information about LR (e.g., LR checklists or baseline assessment; LR statistical registers) in most countries would hinder their practical application. Indeed, having robust spatially implicit baseline data is a prerequisite for any threat assessment or LR monitoring. Then why rely on such data when the costs associated with genomic analysis are becoming less expensive? The reason why biodiversity and LR threat assessment is not done routinely using genomics is the sheer number of taxa or landraces that exist. FAO (2010) estimates there are about 7,000 crops cultivated routinely globally but there is no estimate we are aware of for the number of existent LR, but for rice alone, there are estimated to be approximately 120,000 LRs (Das et al., 2013), though this is probably a high number for a major crop. Even so, an estimate of a total number of over 400M LRs could exist, and routine threat assessment of this large of a cohort using molecular techniques is unrealistic.

As outlined, there have been several diverse attempts to propose a method to threat assess LR material, which in itself demonstrates the urgent requirement for such a method to aid LR conservation planning and maintenance. However, none has been widely applied in practice. Therefore, here we bring together some of the previous LR threat assessment authors and together propose a novel standardised and quantitative method that can be applied to objectively assess LR threat risk at any geographic level or crop.

## 2 Landrace threat assessment methodology

### 2.1 Pre-threat assessment

For LR threat assessment, the unit to be assessed is a LR, but here are preliminary issues that need to be resolved prior to making the actual assessment. These issues are often associated with gathering the necessary information that the assessment is based upon. Depending on the LR to be assessed, much information may already exist, and the process is primarily collation, but for other LRs it may involve generating additional information, commonly time series data related to LR population range, population trend, market and farmer characteristics, and cultivation context. It is also the case that assessment for either Red Listing or LR threat assessment is iterative, meaning the assessment is necessarily repeated because the assessment information for a LR changes over time – therefore there is a need to continue to gather assessment information and periodically repeat the assessment.

The process of gathering assessment information and periodically repeating the threat assessment would normally be discussed by a range of potential stakeholders from the LR maintainer/researcher community (= assessment team) with a particular interest in the LR to be assessed. The issues they might discuss and agree on are likely to include:

a. *LR definition*: The assessment team will need to discuss and agree on what constitutes a LR. LRs are difficult to define precisely (Harlan, 1975; Brush, 2000; Negri, 2003; Camacho-Villa et al., 2005; and Negri et al., 2009). Zeven (1998) believed they were impossible to define, while agreeing they existed, and their conservation was a priority. However, a pragmatic working definition was proposed by Maxted et al. (2020) that a LR is a dynamic population of a cultivated plant species that has a: distinct diagnostic identity (defined in terms of pheno- and genotypic expression), historical origin, not been formally bred recently (with at least 10 generations post initial varietal release), and is also commonly intrinsically genetically diverse, locally adapted to its geographic location, associated with traditional cultivation systems, and with local cultural associations.
b. *Nomenclatural/phenotypic/genomic distinction:* Practically, further clarification is required between genomic, phenotypic, or nomenclatural distinction: is the LR to be assessed defined on its nomenclatural, phenotypic (morphological), or genomic identity? As an individual LR is not as easily identified as biologically distinct species using phenotypic distinction, genomic techniques would be required to decisively identify the populations that represent a specific LR. However, in practice, this would be excessively expensive to enact for the hundreds of thousands of LR and millions of LR populations that exist and might result in the identification of individual genotypes rather than genetically diverse recognised LRs. Therefore, practically LRs are almost always phenotypically (morphologically) and/or nomenclaturally defined. A group of LR populations share distinct, easily observed, and correlated morphological characteristics and/or are known by a single name. Most often a local community will recognise a distinct LR by its morphological characteristics and then use a local name to distinguish that LR. In which case, we assume the populations that have the same name and share morphological characteristics have a unique genetic identity, which is different from other LRs. It is noted that issues related to how landraces are practically recognised and studied are far from novel, some of the pioneers of genetic resources proposed elaborate scientific methods to use classical taxonomical approaches to describe and define basic units of genetic diversity. For example, the ‘eco-geographical classifications’ suggested by Vavilov (1926); Vavilov (1931) and elaborated by Sinskaya (1969) and Mansfeld (1951).
c. *Choice of assessment unit*: The choice of which LR to be assessed is often expedient; if conservation funding becomes available in a particular region, or an array of LR have breeder required trait(s), or a research project generates sufficient LR population descriptive and management data to facilitate threat assessment, then the LR is assessed and those most threatened can then be prioritised and actively conserved. When choosing which LR to threat assess, it could also be argued that care needs to be taken to avoid bias because (i) LR that are assessed as LC or NT will be preferentially assessed because by definition they are more abundant and more likely to be known to farmers/experts, as is evidenced by IUCN Red Listing (Hayward et al., 2015), (ii) LR that are assessed as VH or HI may also be preferentially assessed because they are known by farmers/experts as rare or threatened and assessors wish their preconception confirmed.
d. *Geographic scope (geneflow)*: It is preferable to assess each LR threat status throughout its range to supply the most comprehensive view of its threat status and avoid the need to replicate threat assessment at separate times by different authors in segments of its range. However, this is not always possible, the assessor may not have knowledge of the full geo-political range of the LR, or they may be professionally limited to working on national LR only so LRs found across national borders would be excluded, or a LR may be found on either side of a barrier to geneflow (e.g., mountains, sea) or germplasm exchange (e.g., different ethnic groups, nationality, or even gender). The critical issue is whether geneflow can or is thought to occur among LR populations – if there is geneflow the LR populations can be assessed as one LR but if there is no geneflow the LR populations should be assessed separately. As such, a LR may be assessed at a multi-national, national, national regional, or more restricted level, but in each case the most appropriate geographic scope for the assessment, or rather associated level of geneflow, needs to be agreed pragmatically by the assessment team based on the information available, particularly incorporating knowledge gained from discussion with those cultivating the LR.

### 2.2 Proposed landrace threat assessment methodology

**Figure 1:**
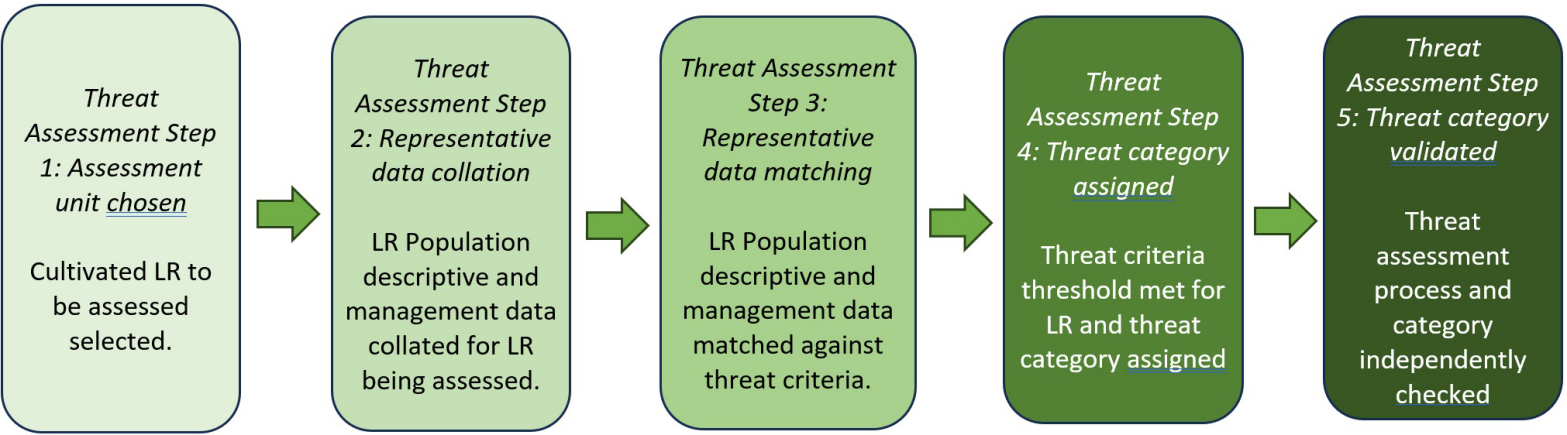
Generalised LR threat assessment procedure.

The LR threat assessment method proposed is in part derived from the IUCN Red Listing method (IUCN, 2001) which is very widely used to assess biodiversity threats and has proven a globally invaluable tool for biodiversity conservation planning, but which is, as argued above, unsuitable for LR threat assessment. Like the IUCN Red List threat assessment so is the LR treat assessment method, but they should not be confused. The generalised principles of both involve five basic steps, but the approach taken is different in its application (**Figure 1**).

*Step 1* − the assessment is focused on a single LR composed of one to many representative populations, a particular LR is selected on the basis of available assessment data and the wish to use the assessment in conservation planning.
*Step 2* − involves the collation of LR representative population descriptive and management data.
*Step 3* − involves the matching of this LR representative population descriptive and management data against the LR threat criteria based on population and range sizes and changes over time, the market and farmer characteristics, and current conservation status. For example, when scoring subcriteria A1.1 LR Geographic Range, the extent of occurrence or area within which the LR population(s) are cultivated is 10km^2^, then a score of 3 would be recorded. This process would be repeated for each subcriteria that data were available and therefore could be scored. The LR threat scores for all the subcriteria scored are summed and the threat percentage is calculated. For example, if scoring a LR 18 out of the 24 subcriteria can be scored, this gives a maximum potential score of 90 (18 subcriteria multiplied by 5, the maximum score for each). Then the actual score for the 18 subcriteria that could be scored is calculated as a percentage of the maximum score possible; in this example 75 out of 90, which is a threat assessment score of 83%.
*Step 4* − the percentage threat score for the criteria that could be assessed is assessed against the threat category threshold and the categories to be assigned for the LR to be assessed is given. If in the example, the threat assessment score is 83% then the LR would be threat-assessed as *Very High (VH)* as the percentage Threat Assessment Score was over 80% for the criteria that could be scored and the LR is facing an extremely high risk of cultivation extinction.
*Step 5* − involves validation, where the threat data, the justification for the threat assessment proposed and the LR threat category proposed summarised in the Assessment Report are checked by a Reviewer in a similar manner to the academic paper standard peer review process. If necessary the reviewer can request changes or approve the LR threat assessment.

**Figure 2:**
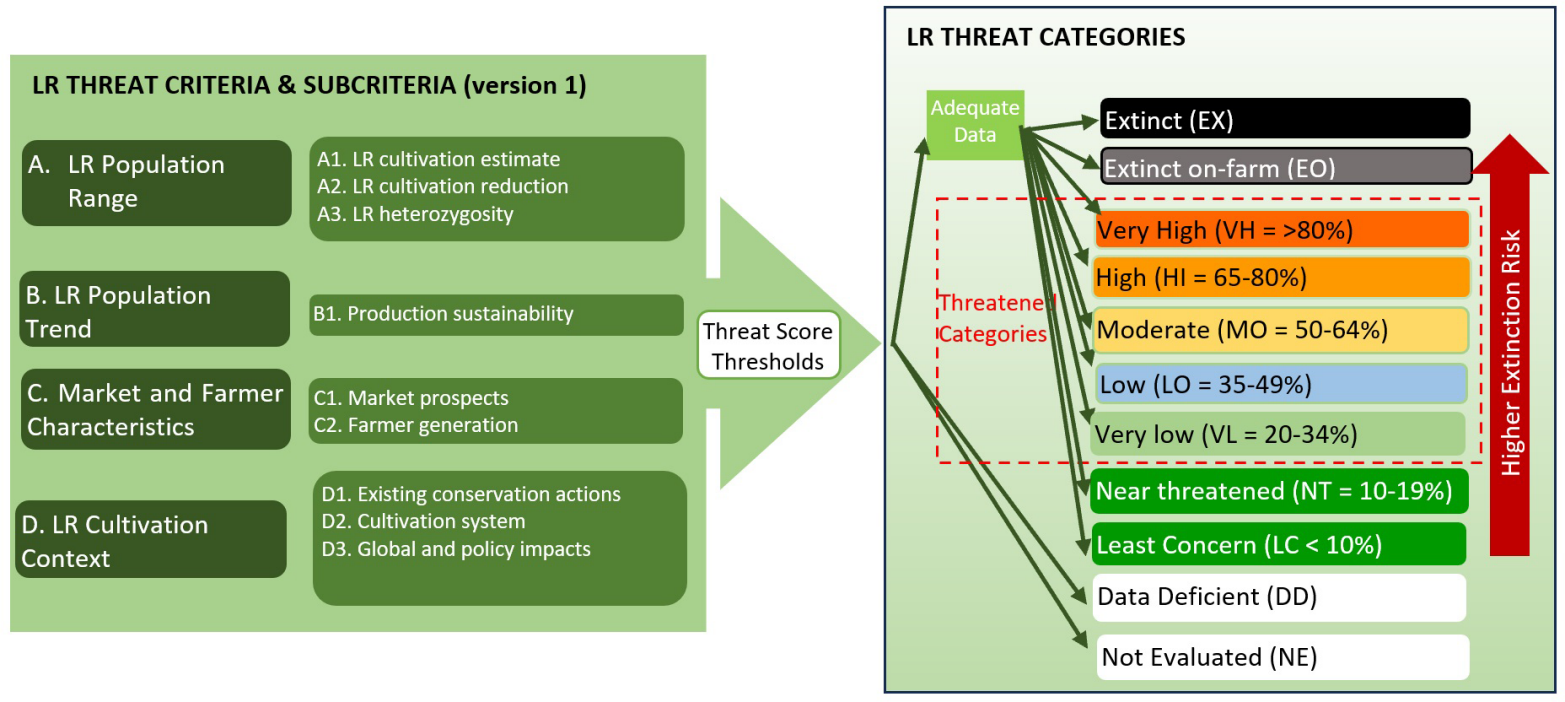
Summary of LR threat assessment criteria and categories.

To acknowledge the link between the Red Listing and LR threat assessment, but also to help avoid confusion between the two approaches, the LR threat categories used are distinct where they are not synonymous with those threat categories used in IUCN Red Listing. Such that the threatened categories for LR assessment are Extinct (EX), Extinct On-farm (EO), Very High (VH), High (HI), Moderate (MO), Low (LO), Very Low (VL), Near Threatened (NR), Least Concern (LC) as well as Data Deficient (DD) and Not Evaluated (NE), as opposed to the IUCN Red List categories (IUCN, 2001, IUCN, 2012) Extinct (EX), Extinct in the Wild (EW), Critically Endangered (CE), Endangered (EN), Vulnerable (VU), Near Threatened (NR), Least Concern (LC), Data Deficient (DD), and Not Evaluated (NE). Both methods use the same terms for the categories Extinct (EX), Near Threatened (NR), Least Concern (LC), Data Deficient (DD), and Not Evaluated (NE), and therefore the definition is identical for both IUCN Red Listing and LR threat assessment as defined here. The definition of the LR unique threat categories is provided in section 2.4 below. See **Figure 2** for a schematic representation of the LR threat assessment process.

### 2.3 Proposed LR threat criteria

LR threat assessment is based on a review of LR descriptive and management information for single or multiple LR populations representative of the LR being assessed. This information is based on available published and grey literature, personal observation of the LR, or focus group meetings with the local communities maintaining the LR. The assessment is based on matching the threat criteria against the characteristics of the LR populations; the criteria are partitioned to indicate a relative threat to LR sustainability and the greater the perceived risk the more likely genetic erosion or extinction.

The threat assessment criteria proposed are split into 4 main criteria, from A to D (A – LR Population Range; B – LR Population Trend; C – Market and Farmer Characteristics; D – LR Cultivation Context), and 24 subcriteria each partitioned to differentiate relative threat. Each subcriteria is divided into relative threat assessment ranges from most (score = 5) to least threatening (score = 1). For subcriteria that cannot be assessed, no score is recorded and they are not included in the threat summary calculation. For an assessment, the scores for each individual subcriteria (5 = most threatened to I = least threatened) that can be scored are summed and then converted to an assessment percentage and this is matched to the LR threat categories, and a category assigned. It may not be possible to score all 24 subcriteria for every LR being assessed, but to ensure that the assessment maintains objectivity it is proposed at least two-thirds of subcriteria are scorable (that is, 16 out of 24 are scorable), if less than two-thirds of subcriteria can be scored then the LR is assessed as Data Deficient.

For several criteria threat is being assessed over time, but what is a scientifically justified time interval to provide meaningful threat assessment in the case of LR? IUCN (2012) in a similar situation uses the last 10 years or three generations, “because measuring changes over shorter time periods is difficult and does not reflect timescales for human interventions” (Mace et al., 2008). However, crops and their LR populations are genetically dynamic, their genetic diversity will change each year, perhaps even more so than wild taxa because they are subject to natural evolutionary pressures, as well as maintainer selection each generation. Even though dynamic change will occur, it will be within limits or the LR itself would lose its distinguishing features. It is also possible that in marginal environments the relative abundance of a particular LR can change substantially year on year, so a 10-year periodicity seems a justifiable timeframe for annual crops. However, it is recognised that this periodicity may need to be amended following further practical application of the LR threat assessment methodology proposed. A further consideration may be the increasing use of citizen science which is likely to facilitate more intense and frequent measurements, if deemed beneficial.

Although practically it is the number of generations that is important not the actual number of years as LR genetic diversity loss can only occur when there is generational change, not within a particular single generation’s lifetime. Further, Jain (1961) investigating the loss of genetic diversity during regeneration found that after 19 generations of bulk composite crossing in annual self-pollinating cereals 50-70% of variation for height and heading was lost but that after 10 generations significant loss of genetic diversity could be detected. Therefore, here the time interval for assessment proposed is over 10 generations, 10 years for an annual crop but longer for a perennial. Ten years may also be thought of as a LR maintainer’s detailed knowledge retention time, about the time a LR maintainer can accurately remember details of the LR they maintain or have knowledge of. It is also recognised that the number of generations may need to be changed when dealing with non-seed-based crops such as those clonally propagated.

The 24 subcriteria are described below and summarised in **Table 1**:

**Table 1:** Version 1 of criteria, subcriteria groups and subcriteria, and indicators of relative threat.

Criteria	Subcriteria	Threat assessment scores					Data sources
		5	4	3	2	1	
A. LR Population Range	A1: LR cultivation estimate						
	*A1.1: Geographic range*	<1 km^2^	1-5 km^2^	6-20 km^2^	21-40 km^2^	≥40 km^2^	Obs.
	*A1.2: Geographic concentration*	<0.5 km^2^	0.5-1 km^2^	2-3 km^2^	4-10km^2^	≥10 km^2^	Obs.
	*A1.3: LR maintainer number*	1	2-5	6-15	16-25	≥26	Obs.
	A2: LR cultivation reduction						
	*A2.1: Geographic range reduction*	≥90%	70-89%	50-69%	30-49%	<30%	Obs.
	*A2.2: Geographic concentration reduction*	≥90%	70-89%	50-69%	30-49%	<30%	Obs.
	*A2.3: Geographic constancy*	≥90%	70-89%	50-69%	30-49%	<30%	Obs.
	*A2.4: Maintainer number reduction*	≥90%	70-89%	50-69%	30-49%	<30%	Obs.
	A3: LR heterozygosity						
	A3.1: LR phenotypic diversity	<30%	30-49%	50-69%	70-89%	≥90%	Farmer Sur.
	A3.2: LR exchange	<30%	30-49%	50-69%	70-89%	≥90%	Farmer Sur.
B. LR Population Trend	B1: Production sustainability						
	*B1.1: Ease of multiplication*	<20%	21-40%	41-60%	61-80%	>80%	Farmer Sur.
	*B1.2: Maintainer continuation*	<30%	30-49%	50-69%	70-89%	≥90%	Farmer Sur.
	*B1.3: LR known loss*	>4	3	2	1	0	Farmer Sur.
	*B1.4: Cultivation of modern cultivars*	90%	70%	50%	30%	10%	Farmer Sur.
C. Market & Farmer Characteristics	C1: Market prospects						
	*C1.1: LR support applied*	No support	–	LR generic	–	LR specific	Farmer Sur.
	*C1.2: Market range*	Local	–	Regional	–	National	Farmer Sur.
	*C.1.3 Food system embeddedness*	Weak (few households)	–	Intermediate (mid nos. households)	–	Strong (most households)	Farmer Sur.
	C2: Farmer generation						
	*C2.1: Maintainer age*	≥70	56-69	41-55	26-40	≤25	Farmer Sur.
D. LR Context	D1: Existing conservation actions						
	*D1.1: Conserved* in situ	No routine maintenance	1-9 pops. on-farm	≥10 pops. on-farm	1-9 pop. conserved	≥10 pops. conserved	Obs.
	*D1.2: Conserved in situ backup*	< 5% pops. duplication	5-30% duplicated	31-70% pops. duplicated	71-95% pops duplicated	>95% pops. duplication	Obs.
	*D1.3: Conserved* ex-situ	No conservation	1-9 pops. conserved	≥10 pops. conserved	1-9 pops. conserved in last 10 yrs.	≥10 pops. conserved in last 10 yrs.	Obs.
	D2: Cultivation system						
	*D2.1: Type of cultivation system*	<30%	30-49%	50-69%	70-89%	≥90%	Farmer Sur.
	*D2.2: Herbicide and fertilizer usage*	≥90%	70-89%	50-69%	30-49%	≤10%	Farmer Sur.
	D3: Global and policy impacts						
	*D3.1: Distorting incentives*	Direct distorting incentives	–	Indirect distorting incentives	–	No distorting incentives	Farmer Sur.
	*D3.2: Global stochastic impact*	≥90%	70-89%	50-69%	30-49%	≤10%	Farmer Sur.

In terms of data sources Obs., Assessment team observation and Farmer Int., Farmer survey.

**A: LR Population Range**

**A1: LR cultivation estimate**

*A1.1: Geographic range* – LR population health is estimated as the geographic spread of a LR estimated using its cultivated extent of occurrence (EOO) (see IUCN, 2012): the smaller the geographic range the greater the LR extinction risk.

*A1.2: Geographic concentration* – estimated as the geographic concentration of the LR using its cultivated area of occurrence (AOO) (see IUCN, 2012): a relatively smaller area of cultivation indicates the relative risk of extinction. A1.1/A1.2 can be assessed using GeoCAT (Bachman et al., 2011) or participatory mapping (Plasencia et al., 2018).

*A1.3: LR maintainer number* – estimated as the number of LR maintainers today repeatedly planting, cultivating, and seed saving: the lower the number of maintainers each going through the cultivation cycle the greater the LR extinction risk.

**A2: LR cultivation reduction**

*A2.1: Geographic range reduction* – estimated as the change in the geographic spread of a LR estimated using its cultivated extent of occurrence (EOO), where the relative decrease in cultivated EOO indicates the relative risk of extinction: the larger the decrease the greater the risk. This is assessed as the average range change over 10 generations. Ten generations is sufficiently long to avoid annual sowing variation being recorded, while permitting distinction of significant long-term changes.

*A2.2: Geographic concentration reduction* – estimated as the change in the geographic concentration of a LR estimated using its cultivated area of occurrence (AOO), where the relative decrease in cultivated AOO indicates the relative risk of extinction: the larger the decrease the greater the risk. This assessed over 10 generations, so 10 years for an annual crop but longer for a perennial.

*A2.3: Geographic constancy* – LR population health is estimated by consistency in cultivation levels (roughly similar areas planted or numbers of plants sown and harvested), in terms of range and concentration assessed over 10 generations, whether maintainers cultivate roughly the same geographic range and concentration for LR generation to generation over the latest 10 generation period. Greater instability of cultivation indicates the rise and fall of LR population levels over time which increases the relative risk of extinction. LR population rise, and fall, is estimated by percentage of population change magnitude (increase or decrease) from generation to generation. Therefore, this is assessed as the average generational change in the LR range and average generational change in LR concentration over 10 generations divided by two.

*A2.4: Maintainer number reduction* – estimated by the relative number of maintainers cultivating LR over 10 generations: reduction in the number of maintainers between the number in year one compared to year ten would be an indication of increased relative risk of extinction.

**A3: LR heterozygosity**

*A3.1: LR phenotypic diversity* – estimated as the amount of phenotypic diversity observed in the LR populations: the greater the diversity the less likely the LR is to be threatened by natural or anthropogenic changes. Phenotypic diversity should be assessed using the standard phenotypic descriptor lists, Bioversity International lists numerous crop-based descriptor lists (https://alliancebioversityciat.org/publications-data), as well as the generalised FAO/Bioversity Multi-Crop Passport Descriptors V.2.1 (Alercia et al., 2015). Here, phenotypic diversity is calculated as the percentage of phenotypic descriptors with at least two or more descriptor states recorded for the LR. Ideally, it is recommended to undertake on-farm characterisation trials with all LR from the region over two cropping seasons, with a minimum of one cropping season. As a minimum the assessment team could interview the maintainers and receive guidance on the relative number of descriptors showing phenotypic variation.

*A3.2: LR exchange* – estimated as the percentage of maintainers that exchange LR material after harvest with other locally-based maintainers. LR material exchange promotes continued heterozygotic diversity and resilience to natural or anthropogenic changes, so reducing extinction risk.

**B: LR Population Trend**

**B1: Production sustainability**

*B1.1: Ease of multiplication* – estimated as the percentage of farmers that report that LR seed/material is abundant and/or potentially easily propagated: relative ease of potential multiplication is an indication of reduced extinction risk.

*B1.2: Maintainer continuation* – estimated as the percentage of LR maintainers that report that within their families or the local community, there is interest in maintaining the LR post current maintainer retirement: the stronger the indication that the next generation of maintainers will continue LR maintenance the smaller the extinction risk.

*B1.3: LR known loss* – estimated as the number of all LR from the same local area known to be no longer cultivated by local maintainers over the last 10 years: the greater the number of LR lost the greater the likelihood that further LR will cease to be cultivated. As above, 10 years may be used as this may be thought of as the LR maintainer’s detailed knowledge retention time, about the time a LR maintainer can accurately remember details of the LR maintained.

*B1.4: Cultivation of modern cultivars* – estimated as the proportion of arable land of the same crop being covered with modern cultivars as the LR being assessed: the greater the proportion of cultivars grown the more likelihood that further LR will cease to be cultivated as maintainers potentially switch to cultivar production.

**C. Market & Farmer characteristics**

**C1: Market prospects**

*C1.1: LR support applied* – identified as any external support (financial or other), primarily from governmental sources, provided to the maintainer or seller that encourages cultivation or marketing of the specific LR being assessed: the presence of LR maintenance incentives indicates reduced threat. Such incentives may be specific, such as particular support for individual LR as recognition under Commission Directive 2008/62 EC, as ‘conservation varieties’ or designation using a quality label, or a regional uniqueness scheme, like the European PDO (Protected Designation of Origin) or PGI (Protected Geographical Indication), which covers agricultural products and foodstuffs. Incentives may also be generic, support for any LR such as Payment for Environmental Services (PES) under the UK Agricultural Bill (UK Parliament, 2020) or the voluntary benefit sharing scheme applied for potato LR in Peru called AGUAPAN, where the private sector directly make payments to LR diversity guardians (see: www.aguapan.org). There are also countries where no specific or generic support for LR maintenance or marketing is provided and here LR are more likely to be threatened with cultivation cessation and extinction.

*C1.2: Market range* – estimated as the breadth of sales and marketing of LRs or LR-derived products in the national, sub-national regional, or local markets: the broader the geographic range of the market for the LR or LR-derived products the less likely the maintainer will cease cultivation. It should be noted that in purely subsistence-based farming systems, there will be relatively low engagement with markets so maintainers will not receive market-based security and are more susceptible to stopping growing LR.

C.1.3 *Food system embeddedness* - estimated as the likelihood of LR use in the regional food system or cuisine: the more LR are embedded in the local cuisine the less likely they are to be threatened. Many LR in purely subsistence-based farming systems may not engage with markets but are conserved at the household level because of their superior quality or organoleptic traits.

**C2: Farmer generation**

*C2.1: Maintainer age* – estimated as the average age of the maintainers that are cultivating and marketing/consuming the LR: the older the maintainer cultivating the LR the more threatened the LR will be as all maintainers must eventually retire.

**D. LR Context**

**D1: Existing conservation actions**

*D1.1: Conserved in situ* – identified by the relative *in situ* on-farm conservation effort: with the most conservation secure LR having more populations actively conserved *in situ* on-farm and the most threatened being those populations of the LR where there is no active on-farm maintenance. Brown and Briggs (1991) suggested that five populations would effectively capture 90-95% common alleles, but this is a minimum number so using 10 populations would aid security of maintenance. Also, here we distinguish between active and passive on-farm conservation, where active on-farm conservation means the maintainer is provided with some form of support to retain existing LR diversity, while passive conservation is where the LR maintainer themselves alone wishes to maintain the LR. Therefore, relatively active on-farm conservation is more secure than passive on-farm maintenance, with a representation of genetic diversity in multiple populations being preferable to a few or single on-farm population, and no regular on-farm maintenance most threatened.

*D1.2: Conserved ex-situ backup* – identified by the proportion of *in situ* populations of the LR sampled and backed up in an *ex-situ* collection: the greater the backup the less likely the LR is to be threatened. It is widely recognised that to be effectively conserved, *in situ* or on-farm populations should be backed-up *ex-situ*. This has two advantages, it means that if the *in situ* on-farm populations are lost they might be reintroduced and restored from the *ex-situ* backup, and the *ex-situ* backup sample might be used to meet any user requirement. As such, it is likely that *ex-situ* backups provide improved chances of survival, as backed-up and used populations are perceived as having higher value and so less threatened.

*D1.3: Conserved ex-situ* – identified by the number and timing of *ex-situ* sampling: with the most conservation secure having higher numbers of LR population and more recent samples conserved as *ex-situ* accessions. To ensure that the genetic diversity in the on-farm populations is relatively well represented in the samples held *ex-situ*, the samples recognised should have been collected and entered the *ex-situ* facility within the past 10 years.

**D2: Cultivation system**

*D2.1: Type of cultivation system* – estimated as the percentage of maintainers with sustainable or traditional farming systems, rather than more commercial or industrial farming systems in the area where the LR is maintained: the greater the number of LR populations maintained within more sustainable or traditional farming systems, the less likely the LR is to be threatened.

*D2.2: Chemical herbicide and fertiliser usage* – estimated as the percentage of maintainers that routinely use chemical herbicides, fungicides, and fertiliser to stimulate production and yield: the greater the proportion of maintainers with LR populations maintained by using more sustainable or traditional farming systems the less likely the LR is to be threatened.

**D3: Global impacts**

*D3.1: Distorting incentives* – Distorting or perverse incentives are benefits provided to LR maintainers by those wishing LR growers to switch to potentially more productive crop varieties. These incentives may be supplied by governments or companies that have a vested interest in promoting cultigen or hybrid production. Distorting incentives may be direct or indirect, meaning they are focused either directly on LR or on the farming system and have an indirect impact on the LR. The more direct the distorting incentives the more likely LR maintainers will switch production and the LR will be eroded or lost.

*D3.2: Global stochastic impact* – estimated as the percentage of maintainers reporting their LR maintenance is being impacted by global deleterious factors such as environmental change, floods, heat, droughts, and wildfires, although these events may be beyond the control of the local community, they can seriously threaten LR maintenance (Jarvis et al., 2010).

### 2.4 Proposed LR threat categories

The LR Threat Categories^1^ used to describe relative LR threat are as follows:

*Extinct (EX)* – A LR is extinct when there is no reasonable doubt that the last population of the LR has been lost on-farm and there are no samples held using *ex-situ* techniques. A taxon is presumed Extinct when exhaustive surveys in known and/or expected regions of cultivation throughout its historic range and *ex-situ* collection surveys have failed to record any cultivated or conserved populations of the LR.
*Extinct on-farm (EO)* – A LR is Extinct On-farm when it is known only to survive in active *ex-situ* conservation, primarily as a seed sample in a genebank, but also possibly as a living plant in a field genebank or seed or tissue culture held in *in vitro* culture or frozen at -196°C in cryopreservation; when exhaustive surveys of previously known areas of cultivation have found no known cultivation either on-farm or in a home garden throughout its historic range it is Extinct On-farm.
*Very High (VH)* – A LR has a Very High risk of extinction when the best available evidence indicates, following LR criterion scoring, that it has a percentage Threat Assessment Score over 80% for the criteria that can be scored, and it is therefore considered to be facing an extremely high risk of cultivation extinction.
High (HI) – A LR has a HIgh risk of extinction when the best available evidence indicates, following LR criterion scoring, that it has a percentage Threat Assessment Score of 65-80% for the criteria that can be scored, and it is therefore considered to be facing a high risk of extinction from cultivation.
*Moderate (MO)* – A LR has a MOderate risk of extinction when the best available evidence indicates, following LR criterion scoring, that it has a percentage Threat Assessment Score of 50-64% for the criteria that can be scored, and it is therefore considered to be facing a moderate risk of extinction from cultivation.
*Low (LO)* – A LR has a LOw risk of extinction when the best available evidence indicates, following LR criterion scoring, that it has a percentage Threat Assessment Score of 35-49% for the criteria that can be scored, and it is therefore considered to be facing a low risk of extinction from cultivation.
*Very low (VL)* – A LR has a Very Low risk of extinction when the best available evidence indicates, following LR criterion scoring, that it has a percentage Threat Assessment Score of 20-34% for the criteria that can be scored, and it is therefore considered to be facing a very low risk of extinction from cultivation.
*Near threatened (NT)* – A LR is Near Threatened by extinction when the best available evidence indicates, following LR criterion scoring, that it has a percentage Threat Assessment Score of 10-19% for the criteria that can be scored, and it is therefore considered to be facing an extremely low risk of extinction from cultivation but is sufficiently close to qualifying for or is likely to qualify for a threatened category in the near future, so the LR should be monitored and reassessed regularly.
*Least Concern (LC)* – A LR is Least Concern when the best available evidence indicates, following LR criterion scoring, that it has a percentage Threat Assessment Score of <10% for the criteria that can be scored, and it is therefore considered to be facing negligible risk of extinction from cultivation. Its cultivation is widespread and locally abundant.
*Data Deficient (DD)* – A LR is Data Deficient when there is inadequate information to make a direct, or indirect, assessment of its risk of extinction based on the available distribution and/or management data. To effectively estimate threat at least two-thirds of subcriteria must be scorable or ≥16 out of 24 are scorable, if less it is assessed as Data Deficient. Listing an LR in this category indicates that more information is required to make an assessment.
*Not Evaluated (NE)* – A LR is Not Evaluated when it has not yet been evaluated against the criteria.

### 2.5 Proposed threat subcriteria data collation

A key component of the LR assessment is collating the data for the assessment subcriteria and, in practice, using a standard questionnaire when interviewing LR maintainers was helpful. The questionnaire was developed from those used by Kell et al. (2009), Fonseca (2004), and the *Banco Português de Germoplasma Vegetal* (BPGV). The data recorded related to the LR maintainer (e.g., farmer’s age, gender); socio-economic conditions; cultivated crops; cultural practices; qualities of LR; and seed characteristics were collected using the questionnaire (see **Table 2**). However, there is also a range of other tools, including quantitative instruments, that can aid the assessment of subcriteria.

**Table 2:** LR threat assessment questionnaire for interviewing LR maintainers.

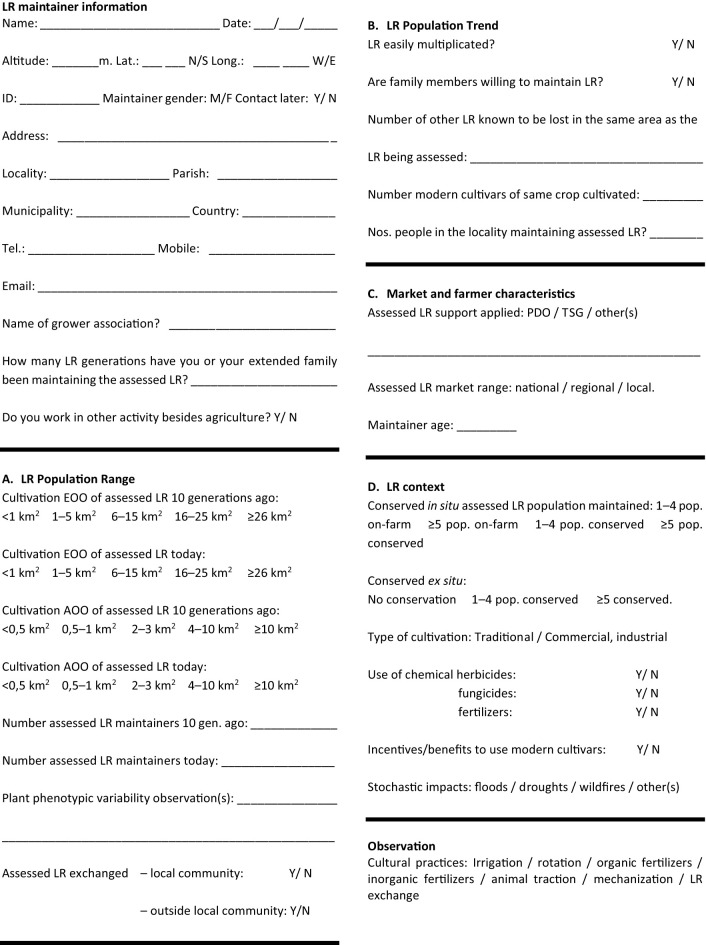

## 3 Discussion

LR diversity is increasingly recognised as a critical resource for contemporary crop improvement (Vavilov, 1957; Frankel, 1970; Frankel, 1972; Harlan, 1972; Bennett, 1973; Hawkes, 1983; Veteläinen et al., 2009; FAO, 2011; Jarvis et al., 2011). Anecdotal evidence and the few LR cultivation reviews undertaken (Veteläinen et al., 2009; Raggi et al., 2022) indicate, despite these resources being a crucial basis for future food security, LR genetic diversity is highly threatened, subject to genetic erosion and extinction, and LR genetic diversity is inadequately conserved therefore unavailable to farmers and breeders for use. However, this general reality differs between LR in crop gene pools and/or geographies (Khoury et al., 2021), and to date there are very few efforts involving systematic PGR *in situ*, even less for LR populations on-farm, monitoring. In this context, it is unnecessarily difficult to plan and conserve LR diversity.

A pivotal factor is the lack of an objective and repeatable method for LR threat assessment is significantly impeding effective conservation planning and implementation, and unavailable LR resources cannot be used (Veteläinen et al., 2009). The intrinsic characteristics of LR, notably the range of diversity/numbers of extant LR, non-standardised nomenclature, lack of comprehensive national LR inventories and the fact that LR populations are maintained by primarily farmers and cultivation is subject to prevailing food systems and market forces, and not conservationists with a single focus on conserving the resource, each makes them a challenging subset of biodiversity to threat assess. The fact that LR conservation focuses on an entirely human-managed resource, not a wild species governed by ecological laws and existing regulatory frameworks, as well as the need to focus conservation at the genetic and not species level, means the straight adaptation of the IUCN Red List method is inappropriate for LR threat assessment and this derived method is urgently needed.

What is presented is a standardised and repeatable method for LR threat assessment derived using the principles that underlie IUCN Red Listing. Initial unpublished case studies testing demonstrates the methodology indicates it is relatively simple to apply, is applicable for multiple crops at multi-national, national, or local levels and would therefore meet the confirmed requirement for an aid to crop and LR conservation planning. However, undoubtedly, the LR threat assessment method proposed requires ground truthing and refinement through actual application on diverse crops in diverse global localities to enhance its value. The current authors are undertaking this task at present. Therefore, it is stressed that what is presented here is version 1 of a LR threat assessment methodology. Just like the IUCN Red List methodology itself it is likely the LR methodology will pass through several revisions following initial practical applications.

In terms of method revision, it is likely that the percentage scores necessary for triggering the seven subcriteria scorable categories and the appropriate time interval for assessment of several of the subcriteria (over 10 generations is proposed here) may need to be revised following practical implementation. Similarly, some subcriteria, such as A2.3 (Geographic constancy), A3.1 (LR phenotypic diversity), and B1.1 (Ease of multiplication) may prove difficult to score practically, if the LR maintainer cannot supply the information needed and those that regularly remain unscorable should be possibly dropped. It is also hoped that practical LR assessments will identify potential additional subcriteria that could be reviewed and possibly added to the methodology. It should also be noted that threat category identification is not the last stage in the process of IUCN Red Listing, once the appropriate category has been proposed the draft Threat Assessment Report (including the category justification) is sent to an independent reviewer to check whether the assessment has been undertaken appropriately and the correct category assigned; ideally the plant genetic resource community should be able to establish a similar review process to mirror the IUCN Red Listing method to ensure scientific objectivity and repeatability.

## 4 Conclusion

The proposed LR threat assessment method presents a first-of-a-kind standardised protocol that can be used globally: in different countries, regions, and with different crops. It would be helpful for the LR threat assessment method to be further evaluated in other global regions and on a full range of crops to see if it is as universal as it currently appears. Nonetheless, the growing LR community interest in developing such a robust threat assessment methodology supports the general need to activate a network for systematic LR monitoring for key crop gene pools globally, to aid their systematic conservation, extend farmer/breeder LR usage and help provide global food and nutritional security.

## Data availability statement

The original contributions presented in the study are included in the article/supplementary material. Further inquiries can be directed to the corresponding author.

## Author contributions

MA: Conceptualization, Investigation, Methodology, Writing – original draft, Writing – review & editing. AB: Investigation, Methodology, Writing – review & editing. SD: Investigation, Methodology, Writing – review & editing. BJ: Methodology, Writing – review & editing. JB: Investigation, Methodology, Writing – review & editing. MY: Investigation, Methodology, Writing – review & editing. NM: Conceptualization, Investigation, Methodology, Writing – original draft, Writing – review & editing.

## Footnotes

1 It is acknowledged that the description of the LR threat categories is derived from the IUCN Red List categories (IUCN, 2001).
